# Consequences of squash (*Cucurbita argyrosperma*) domestication for plant defence and herbivore interactions

**DOI:** 10.1007/s00425-023-04139-7

**Published:** 2023-05-01

**Authors:** Charlyne Jaccard, Wenfeng Ye, Carlos Bustos-Segura, Gaetan Glauser, Ian Kaplan, Betty Benrey

**Affiliations:** 1 Laboratory of Evolutionary Entomology, Institute of Biology, University of Neuchâtel, Rue Emile-Argand 11, 2000 Neuchâtel, Switzerland; 2 Laboratory for Fundamental and Applied Research in Chemical Ecology (FARCE), Institute of Biology, University of Neuchâtel, Rue Emile-Argand 11, 2000 Neuchâtel, Switzerland; 3 Neuchâtel Platform of Analytical Chemistry, University of Neuchâtel, Avenue de Bellevaux 51, 2000 Neuchâtel, Switzerland; 4 Department of Entomology, Purdue University, West Lafayette, IN 47907 USA

**Keywords:** Cucurbitacin, Gene expression, Generalist/specialist, Plant domestication, Plant defence, Root herbivore, Wild relative

## Abstract

**Main conclusion:**

***Cucurbita argyrosperma domestication affected plant defence by downregulating the cucurbitacin synthesis-associated genes. However, tissue-specific suppression of defences made the cultivars less attractive to co-evolved herbivores Diabrotica balteata and Acalymma spp.***

**Abstract:**

Plant domestication reduces the levels of defensive compounds, increasing susceptibility to insects. In squash, the reduction of cucurbitacins has independently occurred several times during domestication. The mechanisms underlying these changes and their consequences for insect herbivores remain unknown. We investigated how *Cucurbita argyrosperma* domestication has affected plant chemical defence and the interactions with two herbivores, the generalist *Diabrotica balteata* and the specialist *Acalymma* spp. Cucurbitacin levels and associated genes in roots and cotyledons in three wild and four domesticated varieties were analysed. Domesticated varieties contained virtually no cucurbitacins in roots and very low amounts in cotyledons. Contrastingly, cucurbitacin synthesis-associated genes were highly expressed in the roots of wild populations. Larvae of both insects strongly preferred to feed on the roots of wild squash, negatively affecting the generalist’s performance but not that of the specialist. Our findings illustrate that domestication results in tissue-specific suppression of chemical defence, making cultivars less attractive to co-evolved herbivores. In the case of squash, this may be driven by the unique role of cucurbitacins in stimulating feeding in chrysomelid beetles.

**Supplementary Information:**

The online version contains supplementary material available at 10.1007/s00425-023-04139-7.

## Introduction

Plant domestication is one of the key developments that allowed the establishment of human societies and modern civilisations (Gepts 2004; Hernandez-Cumplido et al. 2018). All plant structures, including fruits, seeds, roots, and leaves, have been subjected to human selection (Evans 1993) for producing food, fibre, oil, and medicine, among others. For centuries, crops have been transported from their domestication centres to new environments, resulting in diversification and local adaptation, which in some cases was achieved through introgression with the domesticated relatives or with their wild ancestors (Gaut et al. 2015). However, genetic variation tends to be lower in domesticated organisms than in their wild progenitors due to genetic bottlenecks associated with the selection process (Tenaillon et al. 2004; Flint-Garcia 2013; Kates et al. 2021). This loss of diversity varies greatly depending on the species (Innan and Kim 2004; Flint-Garcia 2013) and the purpose of domestication.

The suite of traits that distinguishes a crop from a wild relative is known as the ‘domestication syndrome.’ The genetic basis of ‘domestication syndrome’ may be protein and/or regulatory changes in specific genes, transposable elements, structural variations, or even genome duplications (Olsen and Wendel 2013; Chen et al. 2015a, b). Compared to their wild relatives, genetic changes in crop plants can affect interactions with the plant’s natural enemies, either through changes in the expression of single genes associated with plant resistance or through selection on quantitative traits (Chen et al. 2015a). These changes can result in intraspecific variations in defence compounds in crop plants, either with a complete loss of biosynthesis (Rasmann et al. 2005) or a reduction in secondary metabolites (Poelman et al. 2009), which affect the plant’s interactions with herbivores.

In recent years, there has been increased interest in understanding the impact of plant domestication on their interactions with herbivores and their natural enemies (Turcotte et al. 2014; Chen et al. 2015a, b; Whitehead et al. 2017). Studies on insect oviposition (Idris and Grafius 1996; Bellota et al. 2013), survival (Cardona et al. 1990; Gols et al. 2008), and larval development (Benrey et al. 1998; Szczepaniec et al. 2013) show that increases in herbivore performance and attraction to domesticated plants are often correlated with decreases in plant defence traits (Benrey et al. 1998; Gols et al. 2008; de Lange et al. 2014); however, this pattern is not ubiquitous (Rodriguez-Saona et al. 2011; Turcotte et al. 2014; Chacón-Fuentes et al. 2015; Shlichta et al. 2018; Fernandez et al. 2021). Several factors may be responsible for the lack of correspondence between decreased plant defence traits and increased herbivore performance; plant-related factors, such as the organ targeted by the domestication process or the purpose (Shlichta et al. 2018; Jaccard et al. 2021) and history (Rodriguez-Saona et al. 2011) of domestication, as well as herbivore-related factors, such as the degree of specialisation or mode of feeding (Chen et al. 2015a, b; Whitehead et al. 2017; Gaillard et al. 2018; Shlichta et al. 2018), may play a role.

Most studies on plant domestication and insect interactions have focused on aboveground plant structures (Chen et al. 2015a, b). Comparative studies that examine the consequences of plant domestication on insects that feed on below-ground organs are underrepresented. Only three studies, all in maize, have examined the impact of domestication on below-ground herbivores (Gaillard et al. 2018; Fontes-Puebla and Bernal 2020; Bernal et al. 2022). Although the results of these studies found that insects tended to perform better on domesticated maize, plant genotype and degree of domestication (modern breeding vs local landraces) had strong effects. Thus, to date, there is scarce information on the relationship between altered plant defence traits and the performance of below-ground insects, which would allow comparisons with patterns reported for aboveground herbivores.

Plants from the genus *Cucurbita* have been subjected to several independent domestication events (Nee 1990; Zheng et al. 2013). There are five domesticated species: *C. argyrosperma* Huber, *C. pepo* L, *C. ficifolia* Bouche, *C. moschata* Duschesne, and *C. maxima* Duschesne. All of these species display great diversity in fruit morphology, colour, and purpose of use (Nee 1990). Archaeological records indicate an initial domestication event roughly 10,000 years ago, highlighting that *Cucurbita* is one of the earliest domesticated crops (Smith 1997). Studies on cucurbit domestication date to as early as 1930 (Whitaker and Bohn 1950; Whitaker 1956). Evidence shows that initial domestication of *Cucurbita* was for its use as a container and seed consumption, followed with artificial selection of traits, such as non-bitter flesh and large fruit size (Pickersgill 2007; Zheng et al. 2013). One of the major traits selected during the later process of domestication was the loss or reduction of cucurbitacins, which are extremely bitter and toxic compounds that are even lethal when consumed by many organisms, including mammals (Metcalf 1986; Nee 1990; Balkema-Boomstra et al. 2003). Cucurbitacins have been independently lost from the fruits of all domesticated species (Pickersgill 2018). Selection for non-bitter fruits occurred initially by early humans and later by plant breeders (Rymal et al. 1984; Gry 2006). The loss of fruit bitterness was caused by mutations at the *Bt* locus in cucumber and by mutations in homologous regulators in the syntenic regions of watermelon and melon (Shang et al. 2014; Zhou et al. 2016; Chomicki et al. 2020). In a recent study using two independently domesticated lineages of *C. pepo*, Brzozowski et al. (2020) suggested that the genes responsible for cucurbitacin accumulation in cotyledons are located on the *Bi-4* locus, along with genes relevant for their transport and biosynthesis. However, differences in gene expression associated with chemical defence between domesticated squash and their wild relatives are still unknown.

In a previous study, we compared domesticated varieties of several *Cucurbita* species to examine if the purpose of domestication (fruit consumption/ornamental use) affects plant defences (Jaccard et al. 2021). Results showed great variation in chemical (cucurbitacins) and physical defences (leaf trichomes) among the different varieties, but these were not explained by their domestication purpose. However, the differences found could have been cofounded by phylogenetic differences among the *Cucurbita* species. In the present study, we examined the consequences of squash domestication on plant chemical defence and interactions with herbivores, with wild accessions and domesticated varieties of a single species (*C. argyrosperma*). For this, we used a combination of chemical, molecular, and behavioural analyses. *Cucurbita argyrosperma* is a species of cultural and economic importance locally (Lira-Saade 1995; Barrera-Redondo et al. 2021). We used three wild populations and four domesticated relatives of *C. argyrosperma* that had undergone selection for two different purposes (fruit consumption and ornamental use) and two beetle species, the generalist *Diabrotica balteata* LeConte, 1865 and the squash specialist *Acalymma* spp. (Coleoptera: Chrysomelidae). These plants and insects originate from Mesoamerica, where *C. argyrosperma* was domesticated and where wild and cultivated plants have coexisted for thousands of years (Lira et al. 2016; Sánchez-de la Vega et al. 2018).

In this study, we explored the following questions: (1) How has the domestication of *C. argyrosperma* altered cucurbitacin content (its main chemical defence)? (2) Are the genes in the cucurbitacin metabolic pathway differentially expressed in domesticated varieties? (3) How does altered cucurbitacin content in domesticated varieties correlate with patterns of host-plant preference and performance in generalist and specialist root herbivores? By answering these questions, we tested the hypothesis that domestication affected the pattern of gene expression for cucurbitacins, resulting in reduced chemical defence in domesticated varieties, particularly in those used for consumption humans. Accordingly, we expected that the well-adapted specialist herbivore *Acalymma* spp. would be unaffected by domestication-mediated reductions in cucurbitacin content, while the generalist *D. balteata* would prefer and perform better on domesticated squash. Elucidating the genetic basis of defence traits in wild and domesticated plants and their consequences for herbivorous insects can shed light on the selective pressures that have moulded the interactions we see today and enrich our understanding of natural selection in the wild. In addition, studying below-ground interactions can help us understand how plant domestication has affected plant performance and plant–insect interactions, and thereby identify targets for crop improvement.

## Materials and methods

### Study system

#### Plants

**Fig. 1:**
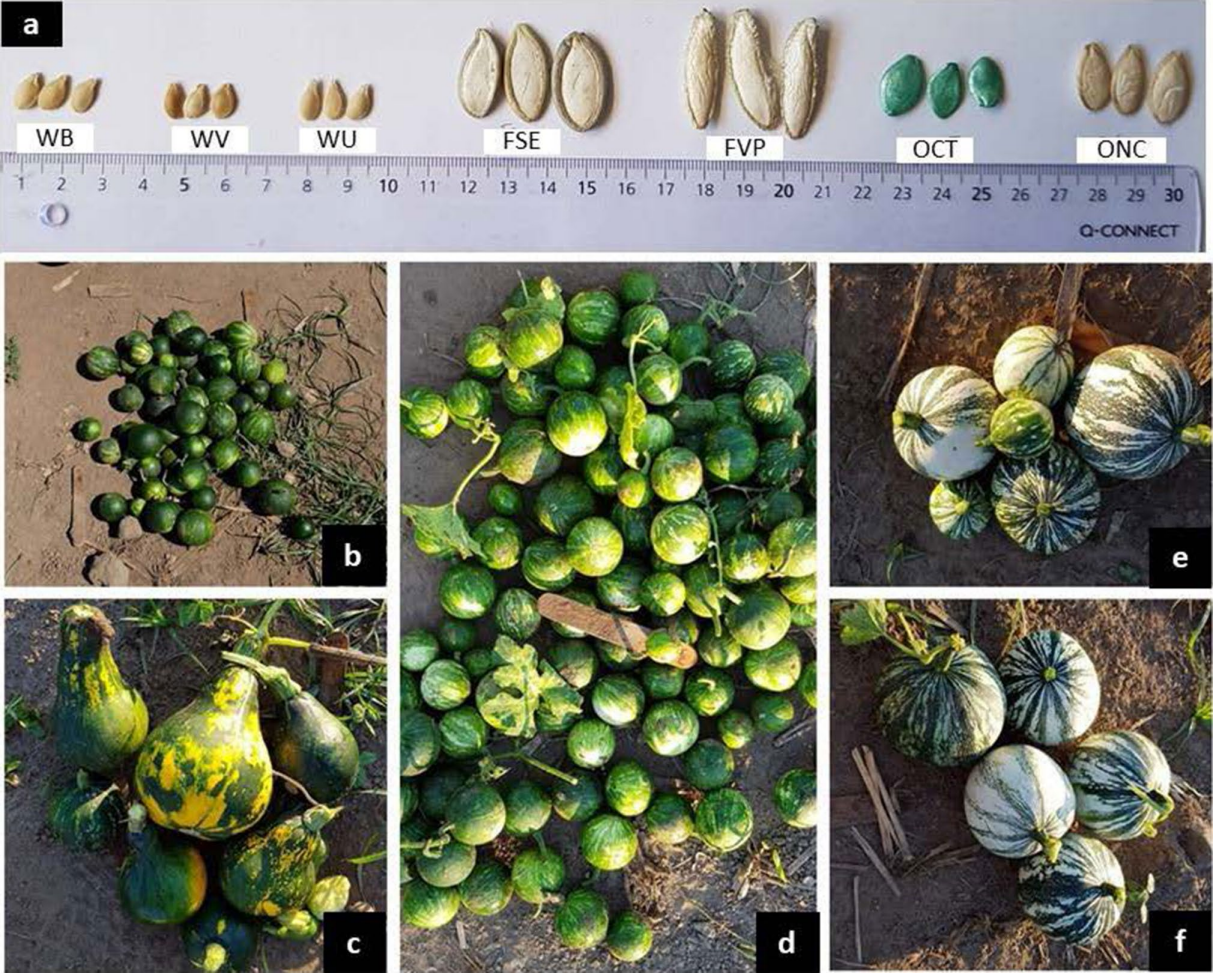
**a** Seeds and fruits of *Cucurbita argyrosperma*. **b** WB: Wild Bacocho. **c** ONC: Navajo Calabacita. **d** WU: Wild Umar. **e** FSE: Silver Edge. **f** FVP: Veracruz Pepita

Wild squash seeds were collected in January 2018 from Puerto Escondido (Oaxaca, Mexico) in the South Pacific coast, where wild *C. argyrosperma* occurs naturally. The climate is hot (average temperature: 27 °C) and humid (84% relative humidity). Fruits were collected from three wild populations along the coast (Wild Umar [WU]: 15°92′49.2″ N, 97°15′09.77″ W; Wild Bacocho [WB]: 15°86′44.6″ N, 97°08′11.4″ W; Wild Ventanilla [WV]: 15°72′79.9″ N, 96°70′86.0″ W). Based on the literature and the fruit shape, the wild plants were identified as belonging to the *C. argyrosperma* species (Jones 1993; Lira et al. 2016; Barrera-Redondo et al. 2021) (Fig. 1b, d). The WV population was used only for cucurbitacin content and insect preference bioassays in the laboratory.

We used four domesticated varieties, including Silver Edge (FSE) and Vera Cruz Pepita (FVP), domesticated for fruit consumption (Fig. 1e, f), and Cushaw Tricolor (OCT) and Navajo calabacita (ONC), domesticated for ornamental use (Fig. 1c). Seeds were obtained from KCB Samen (KCB-Samen GmbH, Bottmingen, Switzerland). Plants were grown with ‘Einheitserde’ classic soil (Einheitsedewerke Werkverband eV, Sinntal-Altengronau, Germany) mixed with 30% sand in plastic pots (8 cm diameter). To enhance the germination of wild seeds, we pierced and scratched the seed coat and placed them between two layers of wet cotton at 28 °C for 1 week. The plants were grown in a greenhouse at ambient temperatures (24 ± 5 °C) under natural light conditions (16:8 h L:D) and were watered as needed. Plants were used for experiments after 15 days of germination, when they had two leaves plus the cotyledons. To control for differences in growth, wild plants were incubated for germination 10 days before the domesticated plants, which grew faster.

#### Insects

*Diabrotica balteata* (Coleoptera: Chrysomelidae), commonly known as the banded cucumber beetle, is an insect pest species which attacks several crops, including cucurbits (Capinera 2001). Adult beetles eat all plant structures, including leaves, cotyledons, and flowers, while larvae feed exclusively on roots and tubers (Capinera 2001). Eggs of *D. balteata* (kindly provided by Syngenta, Stein, Switzerland) were kept in Petri dishes until hatching. Larvae were grown until second instar on maize roots (hybrid DFI 45,321, DSP, Delley, Switzerland), when they were used for experiments. The rearing was kept in quarantine facilities at the University of Neuchâtel (25 °C ± 2 °C, 16:8 h L:D, and 60% RH ± 5%).

The striped cucumber beetle, *Acalymma vittatum* Fabricius, 1775 (Coleoptera: Chrysomelidae: Galerucinae), is a member of the tribe Luperini. This tribe is believed to have an association with plants in the family Cucurbitaceae dating back at least 30 million years (Metcalf 1979; Smyth et al. 2002). *Acalymma vittatum* is considered a specialist; it can consume large quantities of cucurbitacins and is known to metabolise, excrete, and sequester these bitter compounds, which can be present in its body and eggs (Ferguson and Metcalf 1985). Adults feed on leaves, flowers, and pollen, while larvae feed on roots (Eben and Barbercheck 1996; Eben et al. 1997). Laboratory experiments with this species were conducted using a colony maintained in the Insect Ecology Lab at Purdue University, Indiana, USA. Adult beetles were collected locally on cultivated squash and reared on zucchini plants in an incubator (28 °C ± 2 °C, 16:8 h L:D, and 60% RH ± 5%).

### Plant defence measurements

#### Cucurbitacin quantification

Samples of leaves (*n* = 5), roots (*n* = 5), and cotyledons (*n* = 5) of 2-week-old wild and domesticated plants were ground into a fine powder in liquid nitrogen. We weighed 100 mg (± 20 mg) of powder in a microbalance to the nearest 0.1 mg (Mettler Toledo XP6, Columbus, OH, USA) and added 1 mL of methanol (99.99%) (Kaushik et al. 2015) with five glass beads in 1.5 mL Eppendorf tubes. The cells were lysed at 30 Hz for 4 min with a TissueLyser (Qiagen, Hilden, Germany). The lysed samples were centrifuged at 20,913×*g* for 5 min, and 700 µL of surfactant were mixed with 300 µL of MilliQ water. Cucurbitacins were analysed using a UHPLC-QTOFMS instrument with an Acquity UPLC™ coupled to a Synapt G2 high-resolution mass spectrometer (Waters, Milford, MA, USA) at the University Platform of Analytical Chemistry, University of Neuchâtel, as described in Jaccard et al. (2021, 2022). Peaks of known cucurbitacins were automatically integrated using Quanlynx™ with a 0.1 min chromatographic window centred on each component’s retention time and a 0.02 Da mass window centred on the (M + HCOO) ion. All cucurbitacins were quantified by external calibration, with cucurbitacin B serving as a standard. The concentration of cucurbitacin is expressed in micrograms (μg) per gram (g) fresh weight.

#### Real-time PCR (qPCR) for gene expression

RNA isolation was done using the SV Total RNA Isolation System (Promega) following the manufacturer’s instructions. Total RNA of each sample (300 ng) was reverse transcribed using the GoScript™ Reverse Transcription System (Promega). Nine cucumber genes responsible for cucurbitacin biosynthesis (Shang et al. 2014) were used to search for orthologues in *C. argyrosperma* based on its genome database (Barrera-Redondo et al. 2019) using BLAST. Seven candidate cucurbitacin biosynthetic genes (Supplemental Table S1) were found in the genome of *C. argyrosperma.* The qPCR primers were designed using Primer-BLAST (https://www.ncbi.nlm.nih.gov/tools/primer-blast/) and are listed in Supplemental Table S2. We measured differences in the relative expression of these seven genes among the two wild populations (WB and WU, see “Plants” section above) and four cultivated varieties (FSE, FVP, OCT, and ONC). qPCR was performed on a Rotor-Gene 6000 (Corbett Research, Hilden, Germany) platform, using 50 cycles with the following temperature curve: 95 °C for 10 s, 65 °C for 20 s, and 72 °C for 2 s. Five independent biological replicates were analysed, followed with the calculation of the average threshold cycle (Ct) per sample. For the expression analysis of each gene, samples from the WB variety were designated as calibrators. *Actin* was used as a housekeeping gene (GenBank accession number: HM594170), and to calculate relative expression levels with the 2^−△△Ct^ method (Livak and Schmittgen 2001).

### Preference and performance of generalist and specialist chrysomelid larvae on *Cucurbita argyrosperma* roots

The preference and performance experiments with the generalist *D. balteata* were conducted in May 2018 at the quarantine facilities of the University of Neuchâtel, Switzerland. Experiments with the specialist *A. vittatum* were conducted in July 2019 in the Insect Ecology Lab at Purdue University, USA.

#### Choice experiment with beetle larvae on roots of wild and domesticated plants of *C. argyrosperma*

Beetle larval preference for domesticated varieties or wild populations of *C. argyrosperma* was tested in two-choice experiments. Larvae were reared on maize roots (for *D. balteata*) or zucchini roots (for *A. vittatum*) for nearly 10 days until the second instar. We placed the roots in square Petri dishes (12 × 12 cm, Sarstedt, Nümbrecht, Germany) with moist filter paper and a wet cotton ball. For *D. balteata*, we performed two-choice tests, FSE vs WB and OCT vs WV. For the test with *A. vittatum* larvae, only the combination FSE × WB was used, representing the two extremes in cucurbitacin content.

Five *D. balteata* or *A. vittatum* larvae (starved for 12 h) were released in each Petri dish. Petri dishes were then sealed with parafilm to avoid larvae escaping and were covered with red cellophane to reduce higher frequency light and allow observations (Fig. S1). The larvae could move freely to feed on the roots. Larval choice, defined as the number of larvae found on the roots (zero to five), was recorded at 10 min, 30 min, 1 h, 3 h and 24 h. Twenty-two replicates for the OCT × WV combination, 12 replicates for the FSE × WB combination for the experiment with *D. balteata,* and 12 replicates for FSE × WB for the experiment with *A. vittatum*, were performed. Larval choice was calculated and presented as a percentage [(larvae number on roots/5) × 100].

#### Performance of beetle larvae on roots of wild and domesticated *C. argyrosperma*

The performance of *D. balteata* and *A. vittatum* larvae was determined on the roots of wild and domesticated plants. Five-second instars were randomly assigned to a plant of each variety (FSE, FVP, OCT, and ONC) and wild population (WB and WU). Larvae were placed on the roots and soil of 15–21-day-old squash plants inside a small plastic bag. To calculate the initial larval weight, the five larvae were weighed together before the experiment, and the total weight was divided by five to estimate the individual mean weight. The larvae were collected 6 days post-release. The final weight was calculated by weighing all the larvae together for each plant and dividing the total weight by the number of larvae recovered from each bag. Plants were watered with 20 mL tap water once in the middle of the bioassay. The experiment with *D. balteata* was repeated three times with five replicates per variety per experiment (15 replicates in total per variety) and twice (10 replicates) with *A. vittatum*. The plants were randomly distributed in an incubator (28 °C, 12:12 h L:D). Mean value of the relative growth rate was calculated for each larva using final weight divided by initial weight (Tammaru and Esperk 2007; Lariviere et al. 2015).

### Statistical analysis

Data analyses were conducted in R statistical software (v. 4.02.2, R Foundation for Statistical Computing, Vienna, Austria). For all models, we used the Shapiro–Wilk test to determine normality. Cucurbitacin concentrations were analysed separately for each plant tissue (leaves, cotyledons, and roots) to test differences among wild populations and domesticated varieties using the Kruskal–Wallis test. The Wilcoxon rank-sum test with a continuity correction was used for pairwise comparisons.

Choice test of insect preference was analysed for each time point and factor combination with a generalised linear model (GLM) with a binomial distribution. Alpha values were adjusted with Bonferroni correction to correct for multiple comparisons. The relative growth rates (RGR) of *D. balteata* and *A. vittatum* were log-transformed and analysed using a linear model (LM) with variety/population as explanatory variables. Another model was used to test the overall effect of domestication status on the larval RGR with a generalised linear mixed model (LMM), including domestication status as a fixed factor and variety/population as random factors.

For the gene expression experiment, we used GraphPad Prism 8.4.3 and R software. The Kruskal–Wallis test followed with Dunn’s multiple comparisons test were used to compare the differences in gene expression in the roots of wild populations and among varieties. To test the impact of domestication status (wild or domesticated), we used a linear mixed model that included varieties as a random factor.

To test the differences in gene expression between plant tissues (roots vs cotyledons) of the same wild population or domesticated variety, we evaluated the data for homoscedasticity using the *F* test. Normally distributed data with the same standard deviation were analysed using an unpaired *t* test, and normally distributed data with unequal standard deviations were analysed using unpaired *t* tests with Welch’s correction. Non-parametric Mann–Whitney test was used for non-normally distributed data.

## Results

### Wild squash has higher cucurbitacin content than domesticated squash in roots and cotyledons

**Fig. 2:**
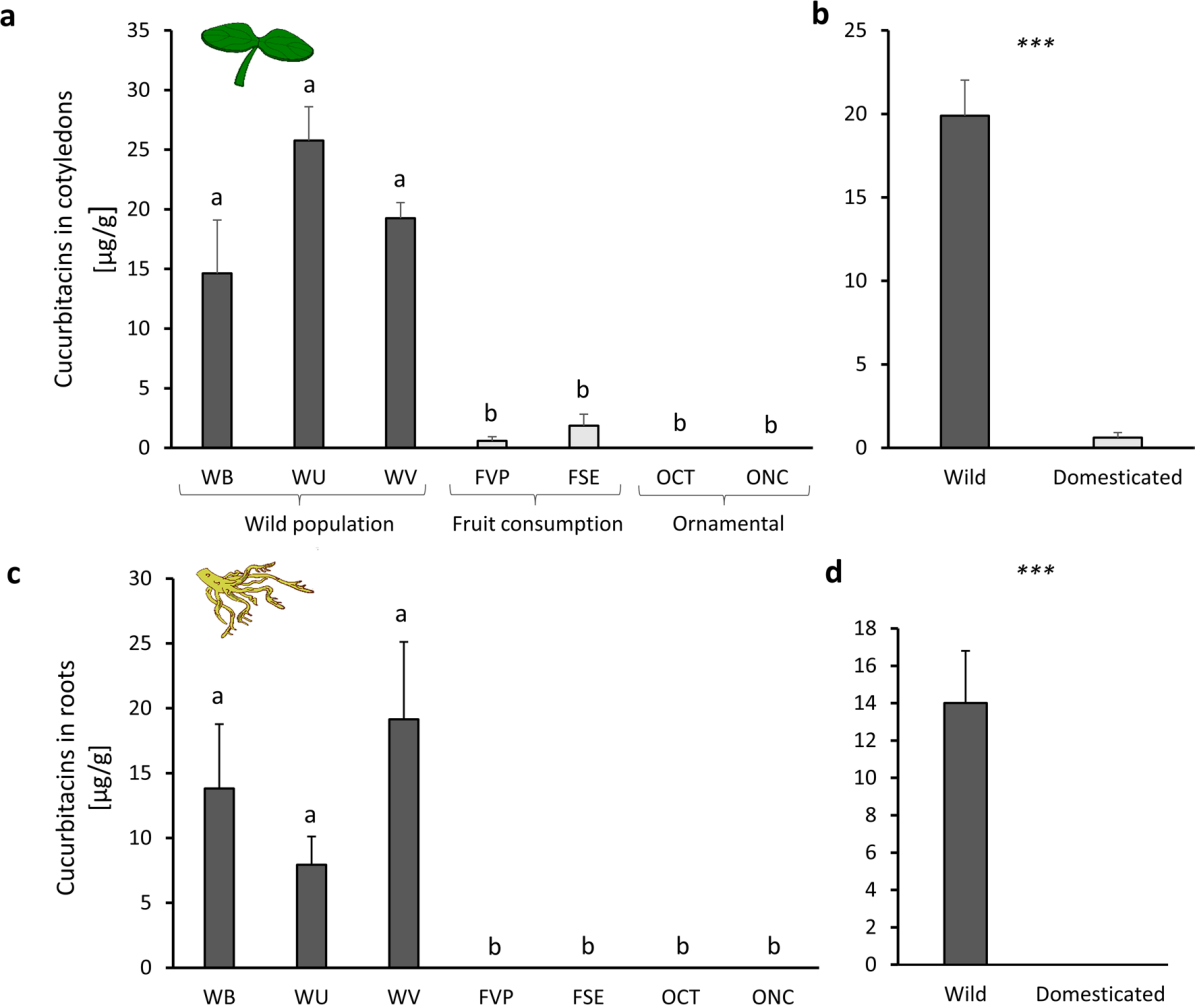
Cucurbitacin concentrations in cotyledons (**a** and **b**) and roots (**c** and **d**) of three wild populations (WB, WU, and WV, in dark grey) of *Cucurbita argyrosperma* and two varieties selected for the fruit consumption (FVP and FSE, in light grey) and two varieties for ornamental use (OCT and ONC, in medium grey). Bars are means (+ SE, *n* = 4). *P* values are given for treatment comparisons with a Kruskal–Wallis test followed by pairwise comparisons using the Wilcoxon rank-sum test with a continuity correction. Letters indicate significant differences among varieties. *** indicate significant differences between wild and domesticated varieties (*P* < 0.0001)

Cucurbitacin concentrations in the cotyledons were significantly different among the seven plant types (Chisq = 24.038, df = 6, *P* = 0.0005) (Fig. 2a). Overall, domestication status explained the difference in cucurbitacin content in cotyledons (Chisq = 20.801, df = 1, *P* < 0.0001) (Fig. 2b), with wild plants containing considerably higher levels than domesticated plants. No significant differences in cucurbitacin content in cotyledons were found among wild populations (WB-WU, *P* = 0.127; WB-WV, *P* = 0.361; WU-WV, *P* = 0.127) or domesticated varieties (FVP-FSE, *P* = 0.561; FVP-OCT, *P* = 0.086; FVP-ONC, *P* = 0.086; FSE-OCT, *P* = 0.086; FSE-ONC, *P* = 0.086; OCT-ONC, *P* = 1) (Fig. 2a).

Cucurbitacin concentrations in the roots were also significantly different among all plant types (Chisq = 26.805, df = 6, *P* < 0.0001) (Fig. 2c). None of the domesticated varieties contained cucurbitacins in their roots. In contrast, cucurbitacins were present at high concentrations (ca. 5–20 μg/g) in the roots of wild populations. No significant differences in cucurbitacin content were found among wild populations (WB-WU, *P* = 0.264; WB-WV, *P* = 1; WU-WV, *P* = 0.429) (Fig. 2c). When data were pooled as wild or domesticated, domestication status explained the difference in cucurbitacin content in roots (Chisq = 26.418, df = 1, *P* < 0.001) (Fig. 2d). No cucurbitacins were found in the leaves of domesticated varieties or wild plants.

### Cucurbitacin biosynthesis gene expression in wild and domesticated plants

**Fig. 3:**
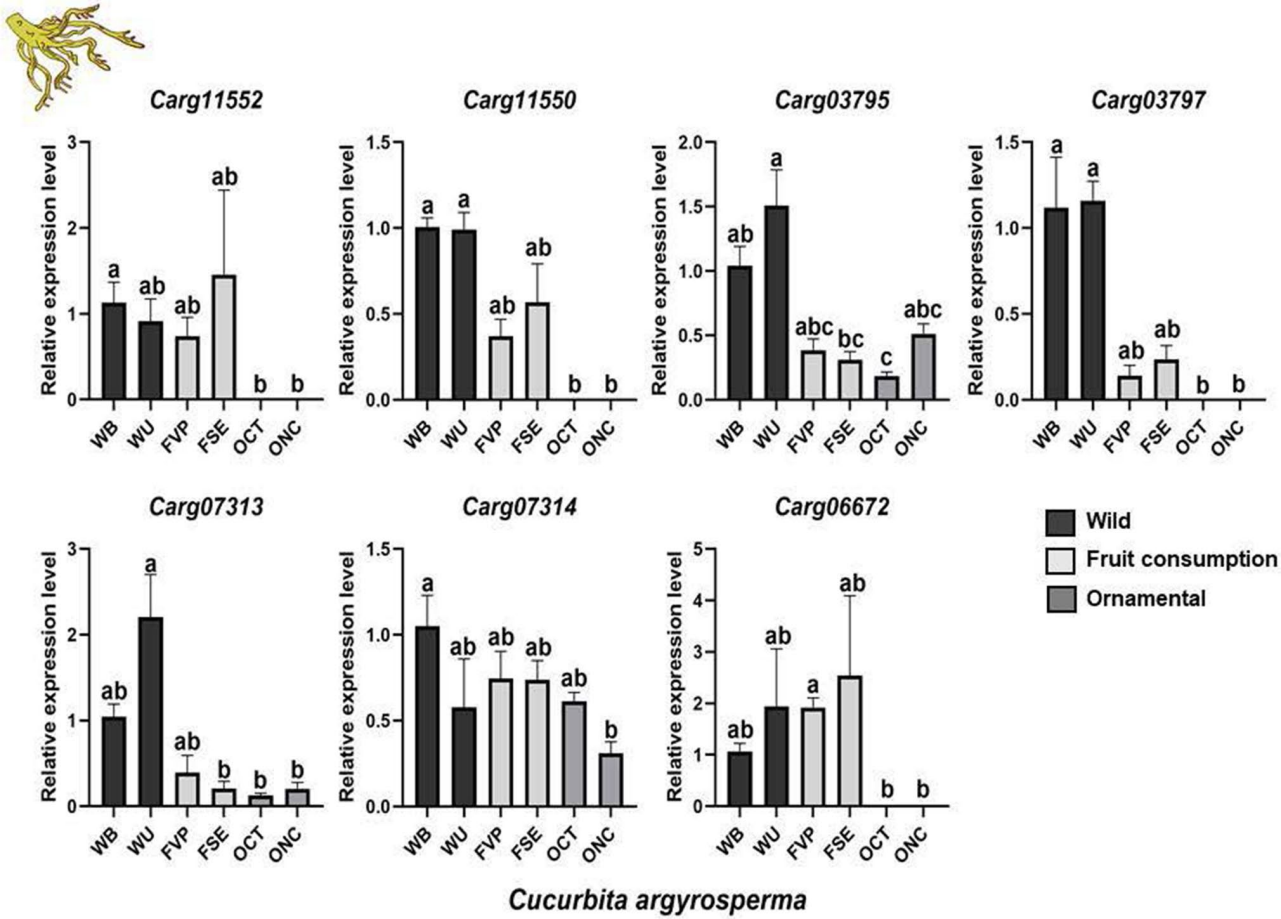
Relative gene expression of seven cucurbitacin biosynthesis genes in roots of two wild populations (WB and WU, in dark grey) of *Cucurbita argyrosperma*, and four related varieties; two fruit consumption varieties (FVP and FSE, in light grey) and two varieties selected for ornamental purpose (OCT and ONC, in medium grey). Shown are means (+ SE). Letters indicate significant differences among wild populations or varieties (*P* < 0.05, Kruskal–Wallis test followed by Dunn’s multiple comparisons test,* n* = 4–6 biological replicates)

*Carg11552* is a candidate *C. argyrosperma* orthologue of the *C. pepo* cucurbitadienol synthase gene, potentially responsible for the first step of cucurbitacin biosynthesis (Shang et al. 2014; Brzozowski et al. 2020). Its expression was higher in the roots of the wild population WB than in those of the ornamental varieties OCT and ONC (Dunn’s test, *P* = 0.0141 and *P* = 0.0427, respectively) but was not significantly higher than in those of the varieties selected for fruit consumption (Fig. 3). The six candidate P-450 cytochrome genes (*Carg11550*, *Carg03795*, *Carg03797*, *Carg07313*, *Carg07314*, *and Carg06672*) showed relatively higher transcription levels in the roots of the wild populations than in the roots of the ornamental varieties (Fig. 3). The FSE variety showed lower expression levels of the genes *Carg03795* and *Carg07313* in the roots than the wild populations. When the data were pooled by domestication status, the relative gene expression was higher in the wild populations than in the domesticated varieties for almost all genes tested (Fig. S2). Surprisingly, the candidate gene coding for the cucurbitadienol synthase (Carg11552*)* was not more highly expressed in the wild populations than in the domesticated varieties (Fig. S2). We also tested the relative expression of these seven genes in cotyledons, but the expression level was almost null in all samples (Fig. S3). Tissue-specific expression analysis revealed that the mRNA levels of *Carg11552*, *Carg11550*, *Carg03797*, and *Carg06672* in the roots of the wild population WB and the fruit consumption variety, FVP, were drastically higher than those in the cotyledons of these plants.

### Generalist and specialist root herbivore larvae prefer to feed on the wild squash

**Fig. 4:**
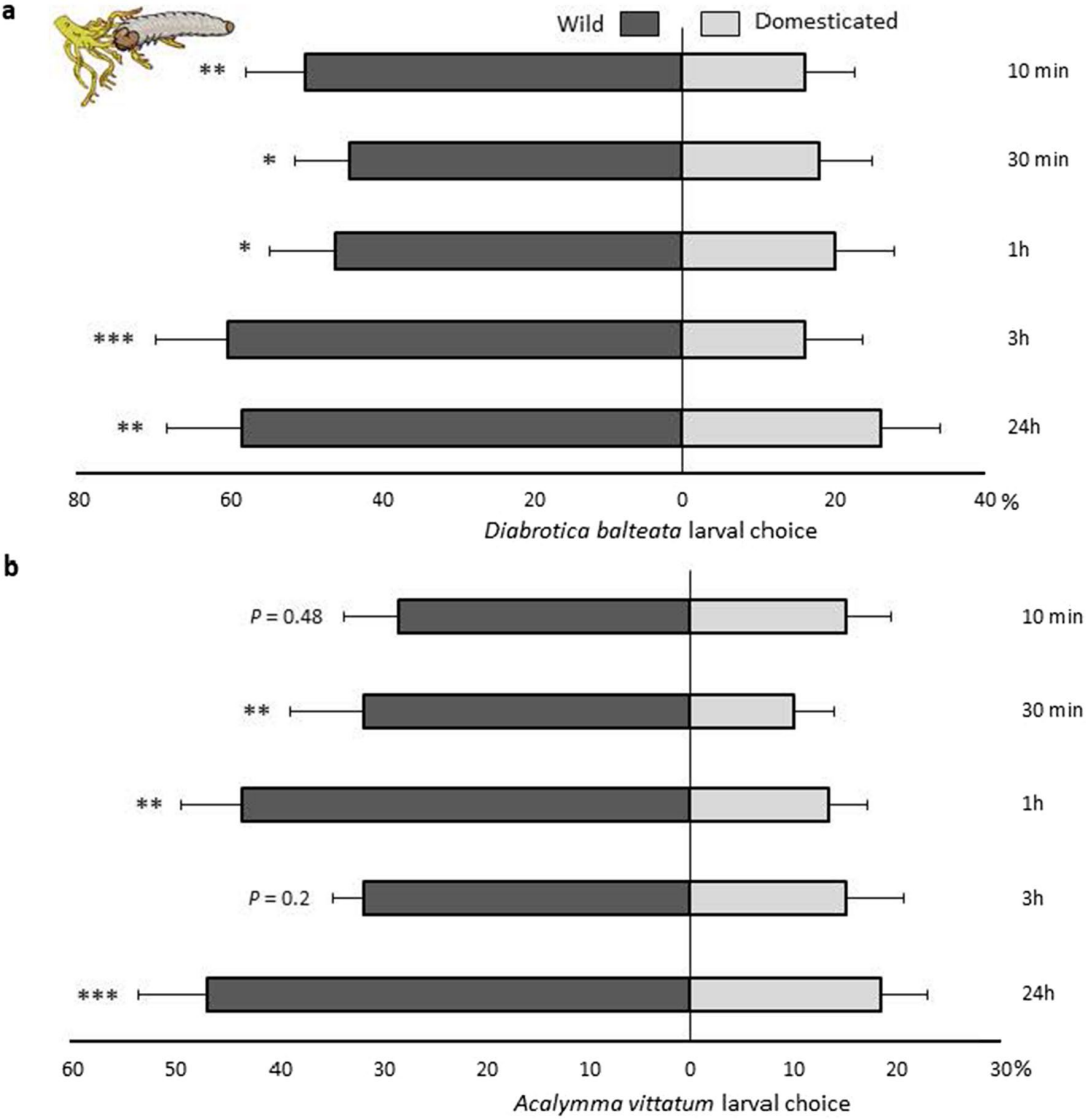
Feeding preference of *Diabrotica balteata* (**a**). The graph represents the mean of the two-choice-test combinations and *Acalymma vittatum* (**b**) larvae when given a choice between roots from the wild population containing cucurbitacins and roots from domesticated varieties without cucurbitacins. Larval choice was measured as the number of larvae on roots after 10 min, 30 min, 1, 3 and 24 h after the experiment began. The bars represent means (+ SE) of the percentage of larvae that chose a plant treatment per time point (y-axis). Treatment comparisons with generalised linear model (family, Binomial) followed by pairwise comparisons of Least Squares Means (LS means) were used and Bonferroni corrected *P* values are indicated with asterisks as follows: * *P* < 0.05, ** *P* < 0.01, *** *P* < 0.001. (**a**: *P* < 0.0005, ANOVA, *n* = 24; **b**: *P* < 0.05, ANOVA, *n* = 12)

Both beetle species (the generalist *D. balteata* and the specialist *A. vittatum*) preferred to feed on wild *C. argyrosperma* (Fig. 4). The results of the two-choice tests for *D. balteata* showed similar results (Fig. S4); therefore, the data were pooled. The strongest preference for *D. balteata* was observed 3 h after the start of the bioassay (z = 4.283, *P* < 0.0001) (Fig. 4a). The specialist *A. vittatum* showed a strong preference for the roots of wild plants at 1 h (z = 3.482, *P* = 0.00249) (Fig. 4b) and 24 h after the experiment began (z = 3.222, *P* = 0.0007) (Fig. 4b).

### Wild squash roots reduce the growth of the generalist *Diabrotica balteata* larvae but not of the specialist *Acalymma vittatum*

**Fig. 5:**
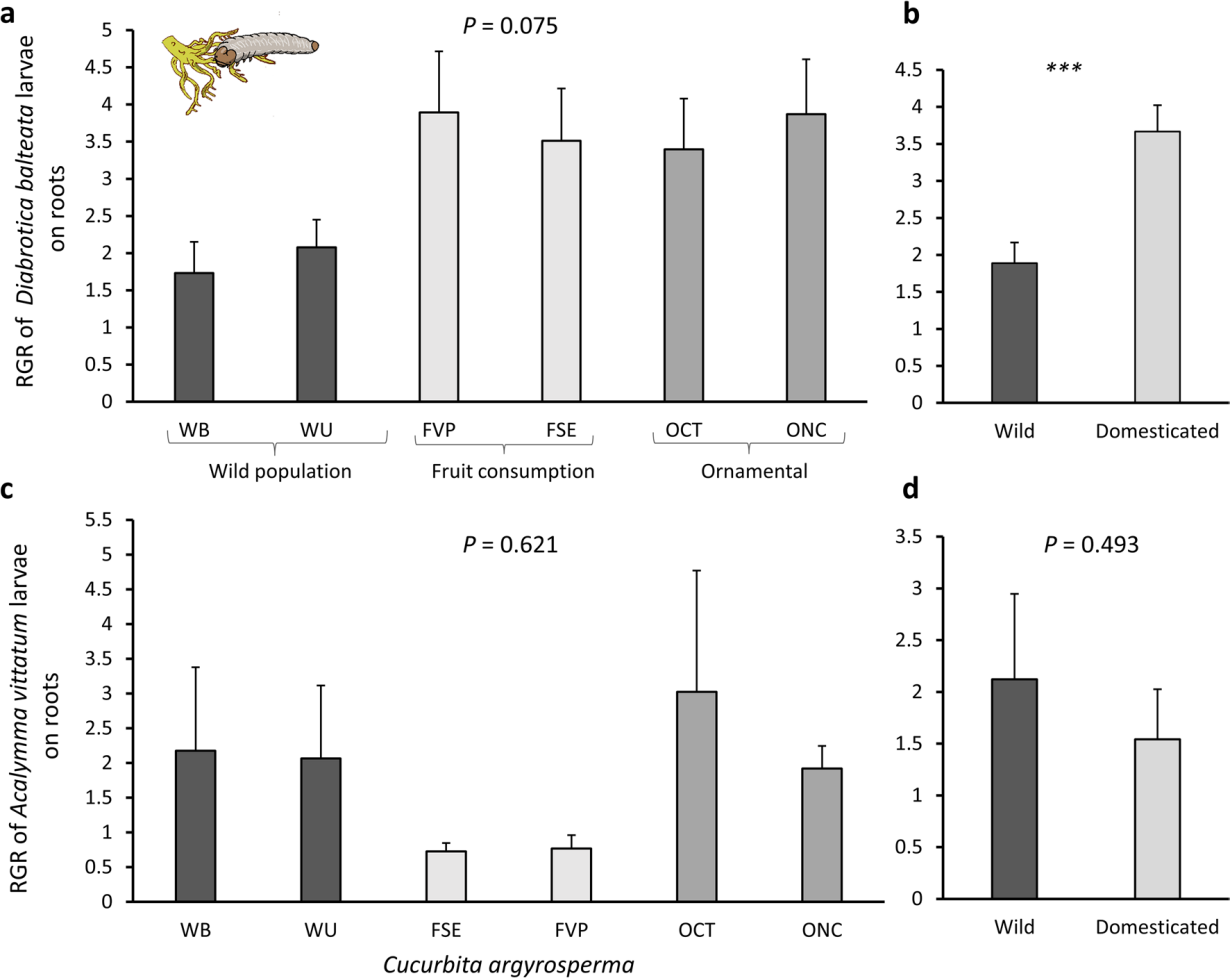
Relative growth rate of *Diabrotica balteata* (**a**) and *Acalymma vittatum* (**b**) larvae fed on *C. argyrosperma* wild population (dark grey) and varieties domesticated for fruit consumption (light grey) and ornamental use (medium grey) after 6 day treatment. (**a**: *P* = 0.075, ANOVA, *n* = 30 for the wild, *n* = 20 for fruit and *n* = 40 for ornamental; **b**: *P* > 0.05, ANOVA, *n* = 5). Bars are means (+ SE). *P* values are provided for treatment comparison with a linear model. Since no significant differences were detected between the groups, data were aggregated based on domestication status, shown in graphs (**b**) and (**d**). ***Indicates significant differences between wild and domesticated varieties

For the generalist *D. balteata*, the growth rate was not significantly different among all plant types (F_5,54_ = 2.14, *P* = 0.075) (Fig. 5a). However, when pooled by domestication status (wild populations or domesticated varieties), larval growth was twofold faster on domesticated varieties, independent of their purpose of domestication (fruit consumption or ornamental use), than on wild plants (Chisq = 10.731, df = 1, *P* = 0.001) (Fig. 5b).

In contrast, the relative growth rate of the specialist *A. vittatum* did not differ significantly among the wild populations or cultivars (F_5,18_ = 0.713, *P* = 0.621) (Fig. 5c) or when pooled by domestication status (Chisq = 0.469, df = 1, *P* = 0.493) (Fig. 5d). However, the larvae grew slower on domesticated varieties than on wild plants.

## Discussion

In this study, we examined how domestication of *C. argyrosperma* altered its chemical and physical defence traits and the consequences for resistance against herbivorous insects. Accordingly, we expected that the pattern of gene expression related to cucurbitacin synthesis would be downregulated in plants from domesticated varieties because of the selection for non-toxic traits, and predicted that the reduction in defence in domesticated plants would increase the performance of a generalist insect but would not affect that of a specialist.

We found support for some of our hypotheses, as well as some unexpected results. Decreasing concentrations of bitter and toxic compounds appears to be an intended goal of the selection process (Nee 1990; Jaccard et al. 2021). As predicted, the expression of cucurbitacin was higher in the roots of wild populations than in those of domesticated plants. However, while varieties selected for ornamental purposes showed no expression of most of the genes linked to cucurbitacin production, resulting in no cucurbitacin synthesis, varieties selected for fruit consumption did express genes involved in cucurbitacin production but did not have cucurbitacins in their roots. These results imply that cucurbitacin biosynthesis in the roots of ornamental varieties might have been inactivated or suppressed during domestication.

The reduction of chemical defences in squash cultivars relative to their wild relatives agrees with most studies on crop domestication (Rosenthal and Dirzo 1997; Rodriguez-Saona et al. 2011; Chacón-Fuentes et al. 2015; Gaillard et al. 2018; Shlichta et al. 2018). The fact that cotyledons of domesticated varieties still contain some cucurbitacins is supported by the ‘optimal defence theory,’ which predicts that highly valuable plant parts should be better defended than older tissues (Zangerl and Rutledge 1996; Turcotte et al. 2014). Injury on cotyledons, the primary photosynthetic organ in seedlings (McCall and Fordyce 2010), would have a high negative impact on the plant performance. Moreover, cotyledons in squash are long-standing tissues that can tolerate a certain amount of herbivory, where the continuous production of secondary compounds could represent an important resource investment (Agrawal et al. 1999).

Numerous research investigated the quantity of genes and mutations necessary for a critical domestication transition (Peleg et al. 2011; Shang et al. 2014; Zhou et al. 2016). A single gene may have played a critical role in domestication transition in many crops (Peleg et al. 2011). The gene expression of cucurbitacins in cucumber was studied by Shang et al. (2014). They identified nine cucurbitacin biosynthetic genes in cucumber, discovered transcription factors that regulate this pathway in leaves and fruits, and found traces of genomic signatures indicating that domestication led to the selection of non-bitter cucumber from its bitter ancestor. In our study, we identified orthologue genes of cucurbitadienol synthase and cytochrome P-450 from the study of Shang et al. (2014), and found the same pattern observed in cucumbers. The expression levels of genes involved in the cucurbitacin pathway were higher in the wild populations than in the domesticated varieties. The lower expression of cucurbitacin C (CuC)-related genes in most of the cultivars was linked to lower production of cucurbitacins relative to the wild populations. This was in agreement with recent results from Brzozowski et al. (2020), who found that in *C. pepo,* biosynthetic genes were expressed in all tissues where cucurbitacins accumulated and not in other tissues, whereas gene expression in cotyledons was undetectable after they were already expanded, even if cucurbitacin was present (Brzozowski et al. 2020). We found that gene expression was higher in roots than in cotyledons (Fig. S3). Since the cotyledons used in our study were already expanded, it is likely that cucurbitacins were synthesised, while the cotyledons were developing, which may explain the expression levels. Thus, gene expression is activated mainly during seedling development and is transient during plant development. An alternative explanation is that enzymes or cucurbitacins are transported from roots to upper plant parts, as observed for alkaloids in other plant species (Pramod et al. 2010). Whether this transport occurs is worth testing in future studies.

We also investigated how traits modified by domestication affected the host preference and performance of generalist (*D. balteata*) and specialist (*A. vittatum*) root herbivore species that commonly attack these plants in the field. We hypothesised that larvae of specialist herbivores prefer bitter plants, as adult beetles are reportedly attracted by cucurbitacins (Eben and Barbercheck 1996; Eben et al. 1997). Our choice experiment showed that the larvae of the specialist and generalist herbivores were more attracted to the bitter roots of wild plants than to those of the domesticated plants. However, *D. balteata* larvae feeding on high-cucurbitacin roots of wild plants show a lower performance. This result reveals that with squash, *D. balteata* behaves like a specialist, even if this is only one of the multiple plant species included in its host range (Capinera 2001). The preference for bitter roots has been previously explained by the hypothesis that some chrysomelid beetles sequester cucurbitacins for their own defence (Ferguson and Metcalf 1985). However, evidence for this idea is still inconclusive (Jaccard et al. 2022; Bruno et al. 2022). An alternative, and to date the most plausible, explanation for the attraction of *D. balteata* to cucurbitacins is that these secondary compounds act as compulsive feeding stimulants (Metcalf and Lampman 1989). It has been reported previously that cucurbitacins in leaves stimulate feeding of adult cucumber beetles (Chambliss and Jones 1966; Hoffmann et al. 1996). Moreover, it was demonstrated that when pure cucurbitacin B was applied on the surface of soybean leaves, *Diabrotica* beetles displayed compulsive feeding behaviours on this non-host plant (Metcalf et al. 1980). Whether cucurbitacins can also act as phagostimulants for Diabroticinae larvae remains to be investigated.

In conclusion, we showed that the domestication of *C. argyrosperma* was selected for a decrease in cucurbitacins in the roots and cotyledons via the lower expression of cucurbitacin-related genes. As predicted, lower levels of cucurbitacins in domesticates differentially affected the performance of generalist and specialist herbivores. Interestingly, both herbivores were highly attracted to high-cucurbitacin plants, revealing that, at least for the generalist insect, there may be opposing selective pressures driving the performance of the larvae and patterns of host-plant selection by the adults. Most studies examining the impact of plant domestication on insect herbivores have focused on insects that feed aboveground. To our knowledge, this is the first study specifically designed to examine the consequences of plant domestication on insects that feed belowground. Additionally, our study offers unique insights on the evolutionary trajectories of wild and domesticated plants, along with the natural and human-mediated selective pressures that have shaped the current interactions, through an examination of the plants and insects that coexist in the region of origin and domestication.

### *Author contributions statement*

CJ and BB originally formulated the idea and designed the experiments. CJ performed the laboratory experiments. GC developed the cucurbitacin-extraction method. CJ and CBS analysed the data. WY designed the primers and performed gene expression experiments. IK provided logistic support and guidance during the experiments conducted at Purdue University. CJ and BB wrote the first version of the manuscript, and all co-authors contributed to the final version.

## Supplementary Information

Below is the link to the electronic supplementary material.

Supplementary file1 (DOCX 356 kb)

## Data Availability

All the data are presented in figures, tables, and Supporting Information.

## Abbreviations

FSE: Squash var Silver Edge
FVP: Squash var Vera Cruz Pepita
OCT: Squash var Cushaw Tricolor
ONC: Squash var Navajo Calabacita
WB: Wild Bacocho
WU: Wild Umar
WV: Wild Ventanilla
