# Supplementary material for: Consequences of squash (Cucurbita argyrosperma) domestication for plant defence and herbivore interactions
Source: Planta. 2023 May 1;257(6):106. doi: 10.1007/s00425-023-04139-7 (PMC10151309; doi:10.1007/s00425-023-04139-7)
Supplement: Supplementary file 1 — Supplementary file1 (DOCX 356 kb) [file 425_2023_4139_MOESM1_ESM.docx]

**Supplementary data**

**Table S1** Genes involved in the cucurbitacins pathway in Cucumis sativus and orthologues found in Cucurbita argyrosperma and their functions.

| **Query gene in cucumber** | **Orthologues in squash** | **Putative Function in cucumber** |
| --- | --- | --- |
| *Csa6G088690* | *Carg11552-RA* | oxidosqualene cyclase (orthologue of cucurbitadienol synthase |
| *Csa6G088160* | *Carg03795-RA* | cytochrome P-450 enzymes (P450s) |
| *Csa6G088170* | *Carg11550-RA* | cytochrome P-450 enzymes (P450s) |
| *Csa6G088710* | *Carg03797-RA* | cytochrome P-450 enzymes (P450s) |
| *Csa3G698490* | *Carg07313-RA* | cytochrome P-450 enzymes (P450s) |
| *Csa3G698490* | *Carg07314-RA* | cytochrome P-450 enzymes (P450s) |
| *Csa1G044890* | *Carg06672-RA* | cytochrome P-450 enzymes (P450s) |

**Table S2** Primers used for qRT-PCR.

| **Genes** | **Forward Primer** | **Reverse Primer** |
| --- | --- | --- |
| *Carg11552-RA* | AGATGGAGGGTGGGGTCTAC | TCTCCAAGCAGCCTTAGTGC |
| *Carg03795-RA* | GCCCACTTCTACTGCCACAT | CATCCCCACCCTCAAACCTC |
| *Carg11550-RA* | CCCTTCTCGCAAATGGGTCT | CTAAACATGGCGTGGCGAAG |
| *Carg03797-RA* | AGCCGTCCGTCTGTTGAAAT | TTCAACGGGACCGCCATAAA |
| *Carg07313-RA* | CGAAATTCGCGTTCGGAGAC | TGATCGCTCAACTTGTCGCT |
| *Carg07314-RA* | CGCGCATTATCGCAAACAGA | AGGAGTGTCTCCGGCTGTAT |
| *Carg06672-RA* | CGCTGGCGGTTGCTTATTAC | AGCCAACTGAAGCGTCTCTC |


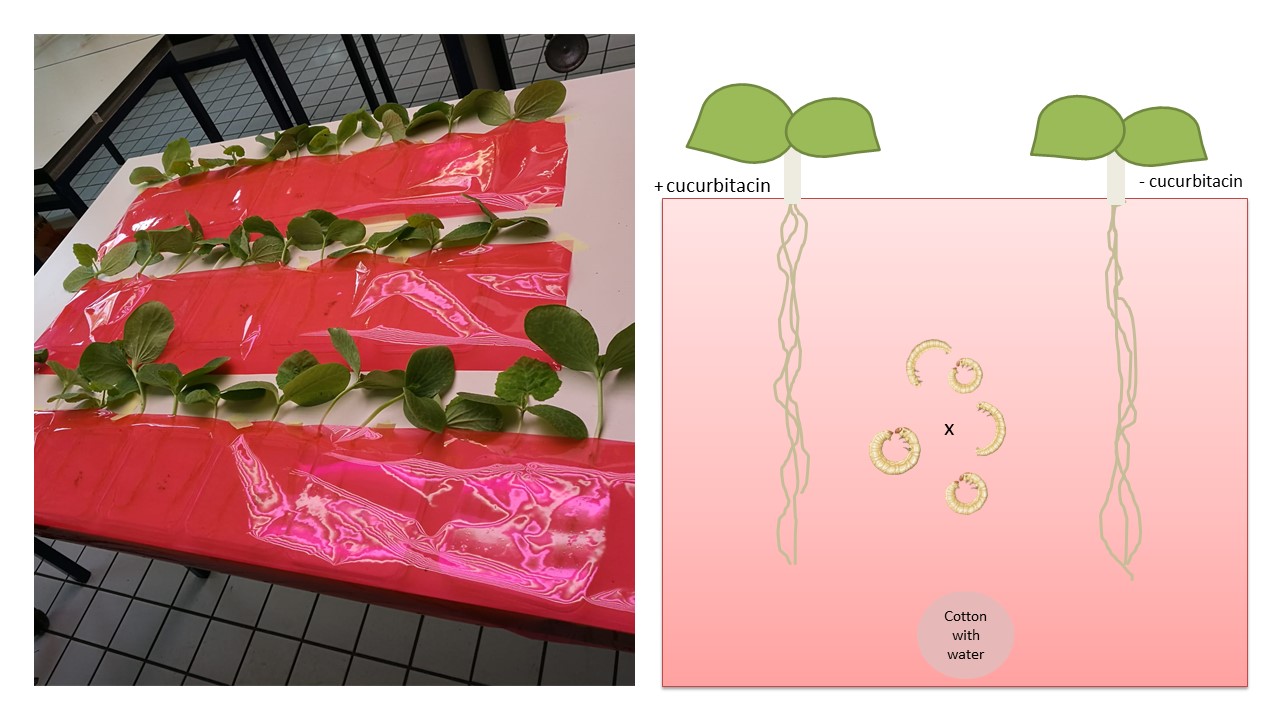
**Fig. S1** Design for the choice-experiment with *D. balteata* on roots of two squash varieties differs in cucurbitacin content. Position of the plant varieties in the squared Petri dishes were alternated. Always five larvae were put in the middle of the petri dish by the hole on the lid. The red cellophane is used to mimic darkness as the UV light cannot go through.


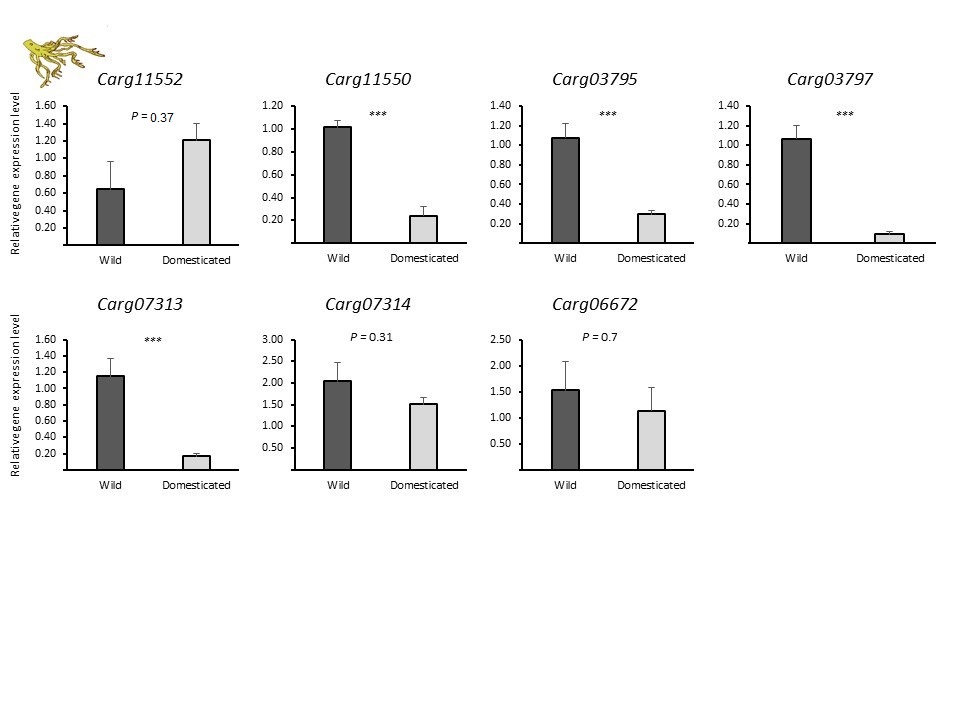


**Fig. S2** Relative gene expression of seven cucurbitacin biosynthetic genes in roots of two wild populations (WB and WU, in dark grey) of *Cucurbita argyrosperma*, and four related domesticated varieties (FVP, FSE, OCT and ONC in light grey). Shown are means (+ SE).

**
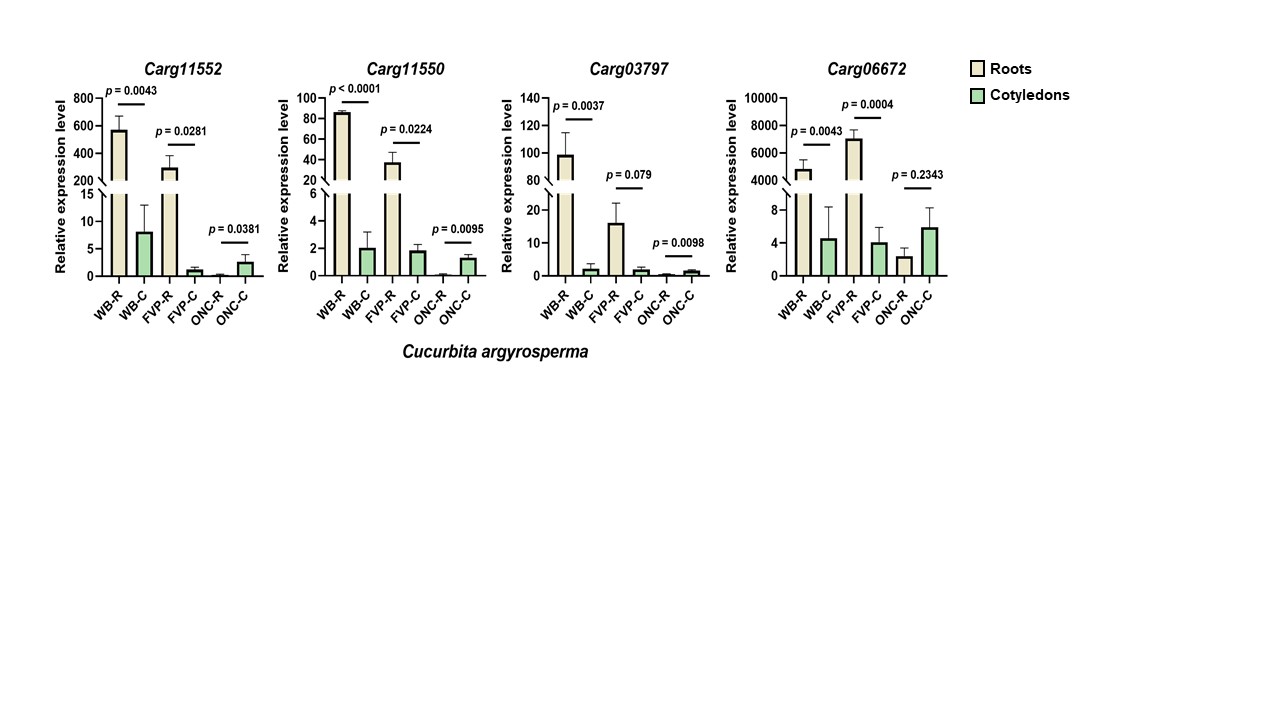
**

**Fig. S3** Relative gene expression levels of four cucurbitacin biosynthetic genes in roots (-R) and cotyledons (-C) of one wild population of *Cucurbita argyrosperma* and two related varieties for fruit consumption (FVP) and for ornamental use (ONC), respectively. Shown are means + SE. *P*-value indicate the difference in gene expression between the two-plant tissue (roots and cotyledons) of the same wild population/variety


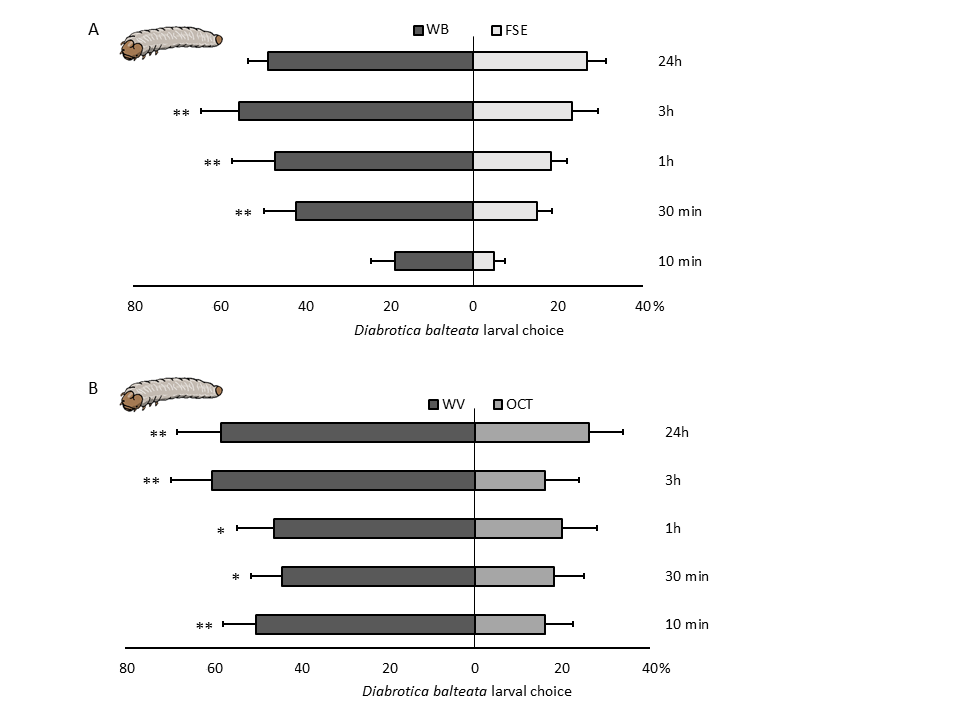


**Fig. S4** Feeding preference of Diabrotica balteata larvae when given a choice between roots from wild population (WB or WV) containing cucurbitacins and roots from domesticated varieties for fruit consumption (FSE) or ornamental use (OCT) without cucurbitacins. Larval choice was measured as the number of larvae on roots after 10 minutes, 30 minutes, 1, 3 and 24 hours after the experiment began. The bars represent means (+ SE) of the percentage of larvae that chose a treatment per time point (y axis). Treatment comparisons with generalised linear model (family, Binomial) followed by pairwise comparisons of Least Squares Means (LS means) were used and Bonferroni corrected P values are provided. * P <0.05, ** P<0.01, *** P <0.001. (**A**: P<0.0005, ANOVA, n=12; **B**: P<0.05, ANOVA, n=22).
